# Modifiable potential risk factors in familial and sporadic frontotemporal dementia

**DOI:** 10.1002/acn3.51619

**Published:** 2022-06-29

**Authors:** Helmi Soppela, Kasper Katisko, Yasmine Gadola, Johanna Krüger, Päivi Hartikainen, Antonella Alberici, Alberto Benussi, Anne Koivisto, Annakaisa Haapasalo, Anne M Remes, Barbara Borroni, Eino Solje

**Affiliations:** ^1^ Institute of Clinical Medicine – Neurology University of Eastern Finland Kuopio Finland; ^2^ Centre for Neurodegenerative Disorders, Department of Clinical and Experimental Sciences University of Brescia Brescia Italy; ^3^ Research Unit of Clinical Neuroscience – Neurology University of Oulu Oulu Finland; ^4^ MRC – Oulu University Hospital Oulu Finland; ^5^ Neuro Center – Neurology Kuopio University Hospital Kuopio Finland; ^6^ Neurology Unit ASST Spedali Civili Brescia Brescia Italy; ^7^ Neuro Center, Neurology Helsinki University Hospital Helsinki Finland; ^8^ A.I. Virtanen Institute for Molecular Sciences University of Eastern Finland Kuopio Finland

## Abstract

**Objective:**

Only a few studies have evaluated modifiable risk factors for frontotemporal dementia (FTD). Here, we evaluated several modifiable factors and their association with disease phenotype, genotype, and prognosis in a large study population including Finnish and Italian patients with FTD and control groups.

**Methods:**

In this case–control study, we compared the presence of several cardiovascular and other lifestyle‐related diseases and education between Finnish and Italian patients with familial (*n* = 376) and sporadic (*n* = 654) FTD, between different phenotypes of FTD, and between a subgroup of Finnish FTD patients (*n* = 221) and matched Finnish patients with Alzheimer's disease (AD) (*n* = 214) and cognitively healthy controls (HC) (*n* = 100).

**Results:**

Patients with sporadic FTD were less educated (*p* = 0.042, B = ‐0.560, 95% CI −1.101 to −0.019) and had more heart diseases (*p* < 0.001, OR = 2.265, 95% CI 1.502–3.417) compared to patients with familial FTD. Finnish FTD patients were less educated (*p* = 0.032, B = 0.755, 95% CI 0.064–1.466) compared with AD patients. The Finnish FTD group showed lower prevalence of hypertension than the HC group (*p* = 0.003, OR = 2.162, 95% CI 1.304–3.583) and lower prevalence of hypercholesterolemia than in the HC group (*p* < 0.001, OR = 2.648, 95%CI 1.548–4.531) or in the AD group (*p* < 0.001, OR = 1.995, 95% CI 1.333–2.986). Within the FTD group, clinical phenotypes also differed regarding education and lifestyle‐related factors.

**Interpretation:**

Our study suggests distinct profiles of several modifiable factors in the FTD group depending on the phenotype and familial inheritance history and that especially sporadic FTD may be associated with modifiable risk factors.

## Introduction

Frontotemporal dementia (FTD) is a clinically, genetically, and pathologically heterogenous group of neurodegenerative diseases characterized by progressive cognitive impairment and alterations in speech, behavior, and personality. FTD is known to be the second most common form of presenile dementia (i.e., dementia with onset before 65 years of age) and the third most common type of dementia in all age groups after Alzheimer's (AD) and dementia with Lewy bodies.^1^

Although most FTD cases worldwide are sporadic, a large number of cases are familial. About 40% of FTD patients have a positive family history of dementia and approximately 10% of these are inherited in an autosomal dominant manner.^2^ The hexanucleotide repeat expansion in the *C9orf72* gene and mutations in *MAPT* and *GRN* genes are the most frequent genetic alterations causing FTD. Of these three, the *C9orf72* repeat expansion is the most common^3^ and especially frequent in the Scandinavia and Northern Europe,^1^ causing the majority of familial FTD and amyotrophic lateral sclerosis (ALS) cases in Finland^4^ while *GRN* mutations are the most common genetic etiologies for FTD in Italy.^5^, ^6^ The *C9orf72* repeat expansion and *GRN* mutations are known to associate with TAR DNA‐binding protein 43 (TDP‐43) neuropathology, whereas *MAPT* mutations lead to hyperphosphorylated tau protein accumulation.

So far, only a few studies have evaluated nongenetic and potentially modifiable risk factors, such as cardiovascular comorbidities or education in FTD. Studies have suggested potential associations between FTD and prior head trauma,^7^, ^8^, ^9^, ^10^ diabetes,^11^ and autoimmune conditions^12^ and conversely, inverse associations between FTD and cardiovascular health.^8^, ^13^ Notably, the cohort sizes of these studies have been limited. Furthermore, the potential associations between these modifiable factors and disease phenotype, genotype, or disease progression in the FTD spectrum are unknown.

Identification of potentially modifiable factors affecting either sporadic or genetic FTD would be crucial for disease prevention and considering potential disease‐modulating treatment strategies.

The aim of the present study was to examine the possible modifiable risk factors, including cardiovascular diseases and education in relation to FTD, and also their influence on disease progression and prognosis in Italian and Finnish patients with FTD. We evaluated several modifiable factors and their association to disease phenotype, genotype, and progression in a large multicenter cohort of Finnish and Italian patients with FTD.

## Materials and methods

### Standard protocol approvals, registrations, and patient consents

The study was performed according to the principles of the Declaration of Helsinki. Written informed consent was obtained from the participants. The study protocol was approved by the research ethics committees of Northern Savo hospital district or Northern Ostrobothnia hospital district and of Brescia hospital.

### Study participants

In total, 1030 patients with FTD participated in the study. Within the FTD group, there were 500 patients diagnosed with behavioral variant frontotemporal dementia (bvFTD), 160 with a nonfluent variant of primary progressive aphasia (nfvPPA), 66 with a semantic variant of primary progressive aphasia (svPPA), 55 with frontotemporal dementia with motor neuron disease (FTD‐MND), 123 with progressive supranuclear palsy (PSP), and 126 with corticobasal degeneration (CBD). Of the FTD cohort, 221 patients were Finns (90/221 from Oulu University Hospital and 131 from Kuopio University Hospital), and 809 patients were Italian (all from Brescia University Hospital). All patients with FTD were diagnosed by a neurologist specialized in cognitive disorders using the latest diagnostic criteria.^14^, ^15^ According to these criteria, three of six features (disinhibition, apathy or inertia, loss of sympathy or empathy, compulsive or perseverative behavior, hyperorality, and dysexecutive neuropsychological profile) are required for a diagnosis of “possible” bvFTD, and “probable” bvFTD adds changes in neuroimaging (frontal and/or temporal atrophy or hypoperfusion) and functional disability. BvFTD with “definite frontotemporal lobar degeneration” requires histopathological proof (autopsy or brain biopsy) or a known causal pathogenic mutation. For PPA patients, a diagnosis requires all three inclusion criteria to be met: difficulties with language as the most prominent clinical feature, daily living activities impaired mainly because of these difficulties, and aphasia as the most significant deficit at the time of symptom onset and during early stages of the disease. Once a PPA diagnosis is made, PPA is further classified into either nfvPPA, svPPA, or lvPPA. Probable CBD criteria require subtle onset and progression of symptoms for at least 1 year, age at onset over 50 years, no notable genetic background (similar family history or known mutations), and a clinical phenotype of probable CBD (motor symptoms). A possible CBD diagnosis has no restrictions on age or family history, allows mutations in tau, and has less strict phenotype fulfillment criteria. Each of the participants underwent appropriate diagnostic procedures including neurological and neuropsychological examination and brain imaging. Most patients underwent genetic testing after giving informed consent. All patients with FTD included in the study fulfilled at least probable bvFTD, nfvPPA, or svPPA criteria^14^, ^15^ or probable criteria for PSP/CBD.^16^, ^17^

As a control group, 214 age‐ and sex‐matched patients with AD were recruited from Finland, 139 from Kuopio University Hospital, and 75 from Oulu University Hospital. All patients with AD were diagnosed by neurologists specialized in memory disorders and the diagnoses were based on neurological and neuropsychological examination, neuroimaging, and cerebrospinal fluid AD biomarker (β‐amyloid_42_, phosphorylated tau, and/or tau protein) testing. All patients fulfilled at least probable AD diagnosis according to criteria by McKhann et al.^18^

The second control group included 100 age‐ and sex‐matched patients with normal cognition recruited and examined at the memory outpatient clinic of Kuopio University Hospital. Individuals in the cognitively healthy control (HC) group were referred to the memory department due to subjective cognitive complaints. They underwent the same evaluations for cognitive disorders as the AD group and were eventually classified as not cognitively impaired and without any diagnosed progressive neurodegenerative disorder at the baseline and after clinical follow‐up. Retrospective evaluation of the electronic patient records was performed until the data collection for this study to exclude any indications of possible neurodegenerative diseases later on.

### Genetic analyses and evaluation of familial inheritance

The *C9orf72* repeat expansion status was analyzed from the FTD cohort using the repeat‐primed polymerase chain reaction assay (Renton et al. 2011). The FTD cohort included 92 *C9orf72* repeat expansion carriers (60 from Finland, 32 from Italy). From the Italian patients, mutations in *GRN*, *MAPT*, and other common FTD‐associated mutations were screened, resulting in 61 *GRN* mutation carriers, 4 *MAPT* mutation carriers, 2 *SQSTM* mutation carriers, 5 *TARDBP* mutation carriers, and 1 *TBK1* mutation carrier. Other genetic alterations than the *C9orf72* repeat expansion were not systematically screened from the Finnish patients, as these mutations have been shown to be extremely rare in the Finnish FTD patients.^19^, ^20^, ^21^ In total, the present cohort included 165 genetic FTD patients with causal mutations.

In addition to the genetic subgroups, we considered the FTD group based on positive family history. The familial FTD group (*N* = 376) included patients with detected causal mutations and/or patients with a Goldman score of ≤3. Sporadic FTD group (*N* = 654) included patients with a Goldman score = 4.^22^

### Clinical review

Medical records of all patients were retrospectively screened for the presence of categorical/dichotomous variables: whether they represented the variable (1) or not (0), at the baseline visit (the cutoff point). The categorical variables included sex, nationality, heart disease (arrhythmias, valvular diseases, coronary artery disease all combined), hypertension, diabetes mellitus, hypercholesterolemia, and smoking (current/ex‐smoker vs. never smoker). As continuous variables, we collected baseline data for Mini‐Mental State Examination (MMSE) and modified FTD clinical dementia rating (FTD‐CDR) scores, years of education, body mass index (BMI), blood total cholesterol concentration, and blood fasting glucose concentration. Family history of neurodegenerative diseases was assessed based on the Goldman criteria.^22^

Only comorbidities present before the baseline visit were included. In cognitively healthy controls, the cutoff point was regarded as the age at which stated cognitively healthy by neurologists based on neurological and neuropsychological examination and other examinations. Age of disease onset was self‐reported by either the patient or a close relative.

The collected variables were compared between the FTD, AD, and cognitively healthy groups (including only Finnish patients in each group) as well as within the total FTD group comparing the FTD subgroups based on familial/genetic status or clinical subtype.

### Statistical analyses

Statistical analyses were performed with SPSS Statistics version 27. The Shapiro–Wilk test and visual inspection were used to evaluate the distribution of the data considering the normality requirements of regression models. Comparison between two categorical variables was compared with binary logistic regression, adjusted for age, sex, and nationality. Two continuous variables or continuous and categorical variables were compared with a general linear model with similar adjustments. A p value of <0.05 was considered statistically significant. Corrections for multiple comparisons were not made due to the exploratory setting of our study, acknowledging the possibility of type 1 error. For each test with a significant p value, also 95% confidence interval was reported to provide information about the range of effect sizes compatible with the data.

### Data availability

Data are available to qualified investigators upon reasonable request with the corresponding author.

## Results

### Characteristics of the whole FTD cohort

Essential characteristics of the whole FTD cohort are shown in Table 1.

**Table 1:** Characteristics of the FTD cohort.

	Finnish FTD (*N* = 221)	Italian FTD (*N* = 809)	All FTD (*N* = 1030)	*p* value with related CI and B/OR values (Finnish vs. Italian FTD)
Age at onset (mean and SD)	60.8* (8.2)	64.1* (8.3)	63.4 (9.0)	*p* < 0.001
Age at diagnosis (mean and SD)	64.4* (11.2)	66.8* (8.3)	66.3 (8.3)	*p* < 0.001
Disease duration, onset to death (years, mean, and SD)	7.6 (5.5)	6.9 (3.8)	7.1 (4.5)	
Phenotype (*N*)				
bvFTD	65.2% (*N* = 144)	14.3% (*N* = 116)	15.5% (*N* = 160)	
nfvPPA	19.9% (*N* = 44)	14.3% (*N* = 116)	15.5% (*N* = 160)	
svPPA	4.1% (*N* = 9)	7.0% (*N* = 57)	6.4% (*N* = 66)	
FTD‐MND	10.4% (*N* = 23)	4.0% (*N* = 32)	5.3% (*N* = 55)	
CBD	0.0% (*N* = 0)	15.6% (*N* = 126)	12.2% (*N* = 126)	
PSP	0.5% (*N* = 1)	15.1% (*N* = 122)	11.9% (*N* = 123)	
Sex, M/F %	51.4	53.0	52.7	
Education (years, mean, and SD)	9.6* (3.3)	8.6* (4.3)	8.8 (4.1)	*p* = 0.021 B = 0.733 95% CI 0.116–1.431
Family history of dementia (GS1‐3) or mutation (%)	52.0	32.3	36.5	
Current or ex‐smoker (%)	24.9*	36.4*	33.6	*p* = 0.001 OR = 0.545 95% CI 0.377–0.788
Heart disease (%)	20.7*	15.0*	16.3	*p* = 0.005 OR = 1.762 95% CI 1.185–2.621
Hypercholesterolemia (%)	55.7*	27.4*	34.0	*p* < 0.001 OR = 3.453 95% CI 2.520–4.733
Cholesterol (mmol/L, mean and SD)	5.0* (1.2)	5.3* (1.1)	5.3 (1.2)	*p* < 0.001 B = −0.399 95% CI −0.610 to −0.189
Fasting glucose (mmol/L, mean and SD)	6.1* (1.2)	5.6* (1.4)	5.7 (1.4)	*p* < 0.001 B = 0.552 95% CI 0.293–0.812
Familial (%)	52.3	32.3	36.6	
MMSE (mean at baseline and SD)	23.5 (4.2)	22.5 (7.3)	22.8 (6.8)	
FTD‐CDR sum of boxes (mean and SD)	5.3 (3.1)	5.8 (4.7)	5.7 (4.3)	

bvFTD, behavioral variant frontotemporal dementia; CBD, corticobasal degeneration; FTD, frontotemporal dementia; FTD‐CDR, frontotemporal dementia‐clinical dementia rating; FTD‐MND, frontotemporal dementia with motor neuron disease; MMSE, MiniMental State Examination; nfvPPA, nonfluent variant of primary progressive aphasia; PSP, progressive supranuclear palsy; SD, standard deviation; svPPA, semantic variant of primary progressive aphasia; *statistically significant difference between Finnish and Italian FTD, *p* < 0.05.

The Finnish and Italian FTD groups had statistically significant differences in age at diagnosis and onset age (*p* < 0.001) and in years of education (*p* = 0.021). Regarding lifestyle‐related factors, the same groups differed in smoking (24.9% in Finnish and 36.4% in Italian patients, *p* = 0.002), fasting glucose (6.1 mmol/L in Finnish and 5.7 mmol/L in Italian patients, *p* < 0.001) and cholesterol levels (5.0 mmol/L in Finnish and 5.3 mmol/L in Italian patients, p < .001), heart disease (20.7% in Finnish and 15.0% in Italian patients, *p* = 0.006), and hypercholesterolemia (55.7% in Finnish and 27.4% in Italian patients, *p* < 0.001). All values were adjusted with sex and age as covariates in the statistical analyses (Table 1).

### Modifiable factors in familial vs. sporadic FTD

Characteristics and comparisons between familial and sporadic FTD groups, considering nationality, age, and sex as covariates, are shown in Table 2.

**Table 2:** Characteristics and comparisons of modifiable factors in familial and sporadic FTD.

	Familial FTD (*N* = 376)	Sporadic FTD (*N* = 654)	*p* value with related CI and B/OR values
Age at onset (mean, years, and SD)	62.0 (9.0)	64.2 (9.0)	
Age at diagnosis (mean, years, and SD)	64.8 (8.3)	67.1 (8.3)	*p* < 0.001
Sex, M/F %	53.1/46.9	52.4/47.6	
Phenotype (*N*)			
bvFTD	54.6% bvFTD (*N* = 206)	45.0% bvFTD (*N* = 294)	
nfvPPA	17.5% nfvPPA (*N* = 66)	14.4% nfvPPA (*N* = 94)	
svPPA	4.0% svPPA (*N* = 15)	7.8% svPPA (*N* = 51)	
FTD‐MND	6.1% FTD‐MND (*N* = 23)	4.9% FTD‐MND (*N* = 32)	
CBD	9.8% CBD (*N* = 37)	13.6% CBD (*N* = 89)	
PSP	7.7% PSP (*N* = 29)	14.4% PSP (*N* = 94)	
Education (mean, years, and SD)	9.3 (4.2)	8.5 (4.1)	*p* = 0.042 B = −0.560 95% CI −1.101 to −0.019
MMSE (mean at baseline and SD)	22.5 (7.2)	22.8 (6.6)	
FTD‐CDR sum of boxes (mean and SD)	6.0 (4.8)	5.5 (4.0)	
Body mass index (mean and SD)	25.8 (4.1)	25.6 (4.4)	*p* = 0.761 B = −0.122 95% CI −0.911 to 0.667
Current or ex‐smoker (%)	30.8	35.5	*p* = 0.257 OR = 1.195 95% CI 0.878–1.628
Heart disease (%)	10.2	20.1	*p* < 0.001 OR = 2.265 95% CI 1.502–3.417
Hypertension (%)	40.9	47.0	*p* = 0.290 OR = 1.162 95% CI = 0.880–1.536
Diabetes (%)	11.3	12.7	*p* = 0.826 OR = 1.048 95% CI = 0.691–1.590
Fasting glucose (mean, mmol/L, and SD)	5.9 (1.5)	5.6 (1.3)	*p* = 0.052 B = −0.241 95% CI −0.484 to 0.002
Hypercholesterolemia (%)	35.0	33.3	*p* = 0.584 OR = 1.085 95% CI 0.810–1.453
Blood cholesterol (mean, mmol/L, and SD)	5.2 (1.1)	5.3 (1.2)	*p* = 0.836 B = 0.021 95% CI −0.177 to 0.219

B, unstandardized beta; bvFTD, behavioral variant frontotemporal dementia; CBD, corticobasal degeneration; CI, confidence interval; FTD, frontotemporal dementia; FTD‐CDR, frontotemporal dementia‐clinical dementia rating; FTD‐MND, frontotemporal dementia with motor neuron disease; MMSE, MiniMental State Examination; N, number of cases; nfvPPA, nonfluent variant of primary progressive aphasia; NS, not significant; OR, odds ratio; PSP, progressive supranuclear palsy; SD, standard deviation; svPPA, semantic variant of primary progressive aphasia.

Patients with sporadic FTD had fewer years of education compared to patients with familial FTD (*p* = 0.042, B = −0.560, 95% CI −1.101 to −0.019). Heart diseases were less prevalent among patients with familial FTD compared with the sporadic FTD group (*p* < 0.001, OR = 2.265, 95% CI 1.502–3.417). No differences were found in BMI, smoking history, and prevalence of hypertension or diabetes mellitus when adjusted for age, sex, and nationality (Table 2).

### Modifiable factors between different FTD phenotypes

Characteristics and comparisons between different FTD phenotypes are shown in Table 3, considering nationality, age, and sex as covariates.

**Table 3:** Characteristics and modifiable factors in clinical subtypes of FTD.

	bvFTD (*N* = 500)	nfvPPA (*N* = 160)	svPPA (*N* = 66)	FTD‐MND (*N* = 55)	PSP (*N* = 123)	CBD (*N* = 126)	*p* value with related CI and B/OR values
Age at onset (mean, years, and SD)	62.1 (9.2)	65.3 (8.4)	62.0 (9.4)	61.2 (9.6)	69.2 (6.4)	62.0 (8.6)	
Age at diagnosis (mean, years, and SD)	65.2 (8.2)	67.4 (8.2)	65.0 (8.1)	64.2 (8.1)	72.4 (6.1)	64.4 (8.3)	
Disease duration, onset to death (mean, years, and SD)	7.5 (4.4)	6.6 (3.3)	8.9 (2.6)	4.9 (7.1)	7.8 (3.4)	6.9 (3.4)	
Sex, M/F %	56.2/43.8	34.4/65.6	45.5/54.5	65.5/34.5	52.8/47.2	59.5/40.5	
Education (mean, years, and SD)	8.6*# (4.0)	9.9* (4.0)	10.4# (4.7)	9.1 (4.1)	7.6 (4.4)	8.3 (3.9)	bvFTD vs nfvPPA *p = <0.001 B = −1.507 95% CI −2.251 to −0.764 bvFTD vs svPPA #*p* = <0.001 B = −1.942 95% CI −2.968 to −0.916
Family history of dementia (GS1‐3) or mutation (%)	41.2	41.3	22.7	41.8	23.6	29.4	
MMSE (mean at baseline and SD)	22.6 (6.3)	19.9 (8.9)	21.2 (7.7)	23.1 (6.5)	24.6 (5.8)	25.2 (4.8)	
FTD‐CDR sum of boxes (mean and SD)	6.5 (4.4)	5.5 (4.1)	5.8 (4.3)	6.4 (4.8)	4.1 (3.5)	3.1 (3.6)	
Body mass index (mean and SD)	27.2 (4.4)	24.4 (4.5)	24.9 (4.5)	24.2 (3.6)	25.7 (4.0)	25.4 (4.2)	NS
Current or ex‐smoker (%)	36.0	27.1	29.6	29.4	35.5	34.3	NS
Heart disease (%)	17.7	16.0	13.1	13.0	19.1	11.4	NS
Hypertension (%)	43.5	37.5	42.6	34.0	62.9	45.7	NS
Diabetes (%)	13.4*	8.3	8.2	11.3	20.9	5.9*	bvFTD vs CBD *p = 0.032 OR = 2.499 95% CI 1.081–5.776
Fasting glucose (mean, mmol/L, and SD)	5.9 (1.4)	5.8 (1.3)	5.4 (0.9)	5.9 (2.1)	5.7 (1.2)	5.4 (1.0)	NS
Hypercholesterolemia (%)	38.2*	29.0*	24.6	41.5	32.2	27.1	bvFTD vs nfvPPA *p = 0.022 OR = 1.654 95% CI 1.076–2.542
Blood cholesterol (mean, mmol/L, and SD)	5.2* (1.2)	5.3 (1.1)	5.6 (1.0)	5.4 (1.1)	4.9* (1.0)	5.5 (0.9)	bvFTD vs PSP *p = 0.036 B = 0.353 95% CI 0.023–0.682

B, unstandardized beta; bvFTD, behavioral variant frontotemporal dementia; CBD, corticobasal degeneration; CI, confidence interval; FTD‐CDR, frontotemporal dementia clinical dementia rating; FTD‐MND, frontotemporal dementia with motor neuron disease; MMSE, MiniMental State Examination; N, number of cases; nfvPPA, nonfluent variant of primary progressive aphasia; NS, not significant; OR, odds ratio; PSP, progressive supranuclear palsy; SD, standard deviation; svPPA, semantic variant of primary progressive aphasia.

*bvFTD vs nfvPPA; #bvFTD vs svPPA.

Patients with bvFTD were less educated than patients with nfvPPA (*p* < 0.001, B = −1.507, 95% CI −2.251 to −0.764) or svPPA (*p* < 0.001, B = −1.942, 95% CI −2.968 to −0.916). Hypercholesterolemia was more common among patients with bvFTD compared to patients with nfvPPA (*p* = 0.022, OR = 1.654, 95% CI 1.076–2.542). In addition, the bvFTD group showed higher prevalence of diabetes mellitus compared to patients with CBD (*p* = 0.032, OR = 2.499, 95% CI 1.081–5.776). Patients in the bvFTD group also had higher blood cholesterol levels compared with patients in the PSP group (*p* = 0.037, B = 0.353, 95% CI 0.023–0.682) (Table 3).

No notable differences in BMI, smoking history, the prevalence of heart diseases or hypertension, and fasting glucose levels were identified between groups when adjusted for age, sex, and nationality. (Table 3).

### Modifiable factors and disease duration in FTD

Regarding the whole FTD group, patients with higher education had a shorter survival from disease onset to death (*p* = 0.023, B = ‐0.148, 95% CI −0.275 to −0.021) with age, sex, and nationality adjustments. However, when genetic status (any causal mutation found) was included as a covariate, no statistically significant differences were found. When calculated separately in groups of *C9orf72* and *GRN* mutation carriers and in patients with presumed TDP‐43 (including *GRN* mutation carriers, *C9orf72* mutation carriers, and FTD‐MND phenotype) vs. tau pathologies (*MAPT* mutation carriers, CBD, and PSP patients), no statistically significant differences were found. Cardiovascular diseases, diabetes, hypertension, and smoking were not associated with disease duration when adjusted for the mentioned covariates. However, in genetic FTD patients (all genetic mutations combined), the presence of prior heart disease is associated with earlier age at onset (56.5 vs. 59.6 years, B = 2.878, *p* = 0.007).

### Modifiable factors in FTD compared with AD and healthy controls

Baseline characteristics of the Finnish study cohort and comparisons between FTD, AD, and HC groups are described in Table 4.

**Table 4:** Characteristics and modifiable factors in Finnish FTD, AD, and control participants.

	Finnish FTD *N* = 221	AD *N* = 214	Controls *N* = 100	Sign. *p* value with related CI and B/OR values
Age at onset (mean and SD)	60.8 (11.2)	64.6 (9.3)	‐	
Age at diagnosis (mean and SD)	64.4 (8.2)	66.8 (8.9)	64.3 (9.8)	
Sex, M %	51.4	51.4	44.0	
Education (years, mean, and SD)	9.6*# (3.3)	10.1* (3.5)	10.3# (3.4)	*p = 0.032 *B = 0.755 *95% CI = 0.064–1.446 #*p* = 0.092 #B = 0.698 #95% CI = −0.114‐1.510
Family history of dementia (GS1‐3) or mutation (%)	52.3	40.2	32.6	
MMSE (mean and SD)	23.5 (4.2)	22.3 (4.2)	26.5 (2.6)	
CDR sum of boxes (mean and SD), FTD‐CDR for FTD patients, AD‐CDR for AD patients	5.3 (3.1)	4.0 (3.8)	‐	
Body mass index (mean and SD)	26.7*# (5.3)	26.3* (5.0)	28.4# (5.3)	*p = 0.830 *B = −0.164 *95% CI −1.672 to 1.344 #*p* = 0.032 #B = 1.681 #95% CI 0.142–3.221
Current or ex‐smoker (%)	24.9*#	27.4*	34.8#	*p = 0.489 *OR = 1.175 *95% CI = 0.744–1.854 #*p* = 0.046 #OR = 1.758 #95% CI 1.009–3.063
Heart disease (%)	20.7*#	25.7*	28.0#	*p = 0.656 *OR = 1.113 *95% CI = 0.695–1.783 #*p* = 0.142 #OR = 1.531 #95% CI 8.867–2.705
Hypertension (%)	44.3*#	48.1*	61.0#	*p = 0.916 *OR = 1.021 *95% CI = 0.688–1.516 #*p* = 0.003 #OR = 2.162 #95% CI = 1.304–3.583
Diabetes (%)	12.2*#	17.8*	19.0#	*p = 0.190 *OR = 1.437 *95% CI = 0.836–2.469 #*p* = 0.109 #OR = 1.693 #95% CI 0.888–3.225
Fasting glucose (mmol/L, mean, and SD)	6.1 (1.2)	6.3 (1.9)	6.1 (1.4)	*p = 0.268 *B = 0.205 *95% CI −0.159 to 0.570 #*p* = 0.843 #B = −0.036 #95% CI −0.389 to 0.318
Hypercholesterolemia (%)	55.7*#	72.0*	77.0#	*p < 0.001 *OR = 1.995 *95% CI = 1.333–2.986 #*p* < 0.001 #OR = 2.648 #95% CI = 1.548–4.531
Blood cholesterol (mmol/L, mean, and SD)	5.0 (1.2)	5.1 (1.1)	5.2 (1.1)	*p = 0.130 *B = 0.185 *95% CI = −0.055 to 0.425 #*p* = 0.314 #B = 0.153 #95% CI −0.146 to 0.451
Disease duration, onset to death (years, mean, and SD)	7.6 (4.2)	8.4 (4.5)	‐	

AD, Alzheimer's disease; B, unstandardized beta; CDR, clinical dementia rating; CI, confidence interval; FTD, frontotemporal dementia; FTD‐CDR, frontotemporal dementia clinical dementia rating; HC, cognitively healthy control; MMSE, MiniMental State Examination; N, number of cases; OR, odds ratio, SD, standard deviation.

*Finnish FTD vs AD; #Finnish FTD vs Controls.

Finnish patients with FTD were less educated (years of education) compared to patients with AD (*p* = 0.032, B = 0.755, 95% CI = 0.064–1.446), whereas there were no major differences found between other groups. The mean total duration of education was 9.6 years in the FTD group, 10.1 years in the AD group, and 10.3 years in the HC group. Patients with FTD were less often smokers/ex‐smokers compared with cognitively healthy controls (*p* = 0.046, OR = 1.758, 95% CI 1.009–3.063). Fewer individuals in the FTD group than in the cognitively healthy control group had hypertension (*p* = 0.003, OR = 2.162, 95% CI 1.304–3.583). In addition, the FTD group showed lower prevalence of hypercholesterolemia than cognitively healthy controls (*p* < 0.001, OR = 2.648, 95% CI 1.548–4.531) or patients with AD (*p* < 0.001, OR = 1.995, 95% CI 1.333–2.986) (Table 4).

There were no major differences in BMI, the prevalence of heart diseases or diabetes mellitus, or fasting glucose and blood cholesterol levels between FTD and AD or cognitively healthy controls when adjusted for age and sex (Table 4).

## Discussion

Here, we have evaluated potentially modifiable factors in both genetic and sporadic FTD by examining associations between cardiovascular burden, education, and FTD. To our knowledge, the previous literature considering environmental factors associated especially with genetic FTD is extremely scarce. Our findings show that patients with sporadic FTD were less educated compared to patients with familial FTD when adjusted for possible confounding factors (age, sex, and nationality). The reason for this remains unclear. The onset of initial symptoms in bvFTD patients usually takes place years before any certain dementia diagnosis is made. Moreover, recent studies have associated FTD with a lifelong neuropsychiatric vulnerability and potential neurodevelopmental problems, suggesting that the disease may manifest even earlier than previously thought, resulting in impaired cognition and learning abilities.^23^, ^24^

Further, the cardiovascular burden was higher in sporadic FTD and especially among patients with the bvFTD phenotype. This finding may result from the fact that patients with causal genetic mutations already carry a greater burden of (genetic) risk factors and are therefore more prone to dementia. Thus, especially sporadic FTD might more likely associate with modifiable risk factors, including cardiovascular diseases and shorter education. Notably, we observed an association between prior heart disease and earlier age at onset in genetic FTD patients, which could indicate that also genetic FTD may be modulated by other environmental factors or comorbidities.

In comparisons between FTD and AD or healthy controls in the Finnish population, FTD group showed a lower prevalence of hypercholesterolemia compared to patients with AD and cognitively healthy controls as well as a lower prevalence of hypertension compared with cognitively healthy controls, suggesting that FTD (as a whole group) may not similarly associate with the cardiovascular burden compared with other types of dementia. Similarly, an Italian study from 2008 showed that cardiomyopathy and hypertension were significantly less prevalent in the FTD group compared with the AD group. However, after adjustments for other variables, the differences were not significant.^13^ In another study from 2012, it was also discovered that the FTD study group was healthier than the control group in terms of heart disease, cerebrovascular disease, and anemia even after statistical adjustments.^8^

The previous literature evaluating potential risk factors of FTD is summarized in Table 5.

**Table 5:** Previous studies reporting associations between FTD and modifiable risk factors.

Study	Country	Participants	Main findings
Rosso, 2003^9^	Netherlands	80 patients with sporadic FTD (no first‐degree relatives with dementia before 70 years of age, no tau mutations found) Control group: 124 patients with normal cognition	Higher prevalence of head trauma in the FTD group (OR 3.3).
Borroni, 2008^13^	Italy	117 patients with FTD, 102 with bvFTD, 15 with temporal variant FTD Control groups: 400 patients with AD, 55 patients with PSP, and 55 patients with CBD	Patients with FTD were more educated than patients with AD. Lower prevalence of cardiomyopathy and hypertension in the FTD group than in AD and PSP groups.
Atkins, 2012^26^	Australia	62 patients with AD Control group: 61 patients with FTD	Higher prevalence of HTA in AD group (OR 2.68). Higher prevalence of smoking in FTD (OR 3.12). Higher body weight in the group (OR 1.03).
De Reuck, 2012^27^	France	22 FTLD brains 19 sporadic, 3 with *GRN* mutation 15 bvFTD, 5 nfvPPA, 1 FTD‐ALS, 1 parkinsonism Control group: 15 healthy brains	Cerebrovascular lesions were rare in FTLD brains, with no difference in prevalence and severity compared with healthy brains (= vascular pathology was not contributing to the disease process). Higher prevalence of white matter lesions in FTLD brains.
Kalkonde 2012^8^	USA	63 patients with bvFTD Control group: 491 patients with non‐FTD dementias: 288 AD, 220 VaD, 46 DLB, and 77 other	Increased risk for FTD in patients with TBI (OR 4.4). Decreased risk for FTD in patients with heart disease (OR 0.4).
Miller, 2013^28^	USA	129 patients with svPPA 39 PGRN mutation carriers Control groups: 186 patients with normal cognition, 158 patients with AD	Higher prevalence of certain autoimmune diseases in svPPA and *PGRN* mutation carriers.
Golimstock, 2014^11^	Argentina	100 patients with FTD Control group: 200 patients with normal cognition	Higher prevalence of diabetes mellitus in FTD.
Deutsch, 2015^7^	USA	1016 patients with FTD, 710 with bvFTD, 154 with nfvPPA, 152 with svPPA Control group: 2015 patients with normal cognition	Higher prevalence of head trauma in FTD (OR 1.67) Patients with FTD were less educated and younger.
Torralva, 2015^29^	USA	62 patients with bvFTD and cerebrovascular disease (V‐bvFTD) Control group: 329 patients with bvFTD without the cerebrovascular disease (NV‐bvFTD)	Lower prevalence of hypertension and higher prevalence of stroke in the V‐bvFTD group. Patients with NV‐bvFTD possibly had a more aggressive neurodegenerative disease.
LoBue, 2016^10^	USA	75 bvFTD patients with previous traumatic brain injury with loss of consciousness 603 bvFTD patients with no previous traumatic brain injuries with loss of consciousness	Patients with previous traumatic brain injury with loss of consciousness had an earlier age of symptom onset and age of diagnosis
Katisko, 2018^30^ Katisko, 2018^12^	Finland	195 patients with FTD Control groups: 193 patients with AD 184 patients with normal cognition	Lower prevalence of cancer in FTD. High prevalence of immunological disorders especially in sporadic FTD.
Westeneng, 2021^31^	Netherlands	143 C9‐positive patients with ALS 1322 C9‐negative patients with ALS Control group: 1322 patients with normal cognition	Lower body mass index, lower physical activity, and increased energy intake in the *C9orf72* carrier group. Increased physical activity in *C9orf72* noncarriers Lower alcohol intake and higher tobacco intake in both ALS groups.

AD, Alzheimer's disease; ALS, amyotrophic lateral sclerosis; bvFTD, behavioral variant frontotemporal dementia; C9, chromosome 9 repeat expansion mutation; CBD, corticobasal degeneration; DLB, dementia with Lewy bodies; DM, diabetes mellitus; FTD, Frontotemporal dementia*; GRN*, granulin; nfvPPA, nonfluent variant primary progressive aphasia; NV‐bvFTD, behavioral variant frontotemporal dementia without cerebrovascular disease; HTA, hypertensio arterialis; OR, odds ratio; PSP, progressive supranuclear palsy; TBI, traumatic brain injury; VaD, vascular dementia; V‐bvFTD, behavioral variant frontotemporal dementia with cerebrovascular disease.

Overall, our findings suggest that a low level of education and cardiovascular health could represent modifiable risk factors in FTD, and that heart health may modulate age at onset in genetic FTD. Heart disease, baseline fasting glucose and blood cholesterol levels, diabetes mellitus, and body mass index do not appear to associate with an increased risk of FTD in general according to our study, and no statistically significant differences were found considering these variables when comparing Finnish FTD groups to AD and HC.

The strengths of this study include our large multinational cohort. Furthermore, we had patients of each clinical subgroup, and the portion of various causative mutation carriers was relatively large, which reflects the heterogenous nature of the FTD spectrum diseases. The limitation of this study is its retrospective setting. In this study, with data from multiple centers, we did not have sufficient comparable data on other dementia‐related modifiable risk factors (alcohol consumption, physical activity, hearing impairment, depression, air pollution, traumatic brain injury, and low social contact according to Livingston et al.^25^) and this may be considered as a limitation. We acknowledge that the multicenter nature of the present cohort may predispose the study to biases related to epidemiological and/or methodological differences in the FTD population or general population between different centers and nations. It should also be noted that although all patients were European, there may still be differences in the FTD risk factor profiles between different countries, as shown in Table 1. In addition, the comparison between FTD patients, AD patients, and healthy controls was performed only in the subpopulation of the study participants, which limits the applicability of the results. To underline the exploratory setting of our study, no corrections for multiple comparisons were made. Thus, our statistical approach is more prone to type 1 error, that is, false‐positive findings, and the p values should be evaluated only alongside the reported confidence intervals.

In conclusion, the modifiable risk factor profile is different in sporadic vs. genetic forms of FTD. Lower education and impaired cardiovascular health are associated especially with the sporadic form of FTD and especially with the bvFTD phenotype. Although patients with familial FTD appear to be relatively unaffected by the studied lifestyle‐related risk factors, a potential association between heart diseases and earlier age at onset in genetic FTD was observed. Further research on the subject is needed to confirm these findings and their role as a potential area of intervention.

## References

[1] Bang J , Spina S , Miller BL . Frontotemporal dementia. Lancet. 2015;386(10004):1672‐1682. doi:10.1016/S0140-6736(15)00461-4 26595641 PMC5970949

[2] Rohrer JD , Guerreiro R , Vandrovcova J , et al. The heritability and genetics of frontotemporal lobar degeneration. Neurology. 2009;73(18):1451‐1456. doi:10.1212/WNL.0b013e3181bf997a 19884572 PMC2779007

[3] DeJesus‐Hernandez M , Mackenzie IR , Boeve BF , et al. Expanded GGGGCC hexanucleotide repeat in noncoding region of C9ORF72 causes chromosome 9p‐linked FTD and ALS. Neuron. 2011;72(2):245‐256. doi:10.1016/j.neuron.2011.09.011 21944778 PMC3202986

[4] Renton AE , Majounie E , Waite A , et al. A hexanucleotide repeat expansion in C9ORF72 is the cause of chromosome 9p21‐linked ALS‐FTD. Neuron. 2011;72(2):257‐268. doi:10.1016/j.neuron.2011.09.010 21944779 PMC3200438

[5] Moore KM , Nicholas J , Grossman M , et al. Age at symptom onset and death and disease duration in genetic frontotemporal dementia: an international retrospective cohort study. Lancet Neurol. 2020;19(2):145‐156. doi:10.1016/S1474-4422(19)30394-1 31810826 PMC7007771

[6] Borroni B , Bonvicini C , Galimberti D , et al. Founder effect and estimation of the age of the progranulin Thr272fs mutation in 14 Italian pedigrees with frontotemporal lobar degeneration. Neurobiol Aging. 2011;32(3):555.e1‐555.e8. doi:10.1016/j.neurobiolaging.2010.08.009 20947212

[7] Deutsch MB , Mendez MF , Teng E . Interactions between traumatic brain injury and frontotemporal degeneration. Dement Geriatr Cogn Disord. 2015;39(3–4):143‐153. doi:10.1159/000369787 25531628 PMC4427348

[8] Kalkonde YV , Jawaid A , Qureshi SU , et al. Medical and environmental risk factors associated with frontotemporal dementia: a case‐control study in a veteran population. Alzheimers Dement. 2012;8(3):204‐210. doi:10.1016/j.jalz.2011.03.011 22465176

[9] Rosso SM , Landweer EJ , Houterman M , Donker Kaat L , van Duijn CM , van Swieten JC . Medical and environmental risk factors for sporadic frontotemporal dementia: a retrospective case‐control study. J Neurol Neurosurg Psychiatry. 2003;74(11):1574‐1576. doi:10.1136/jnnp.74.11.1574 14617722 PMC1738250

[10] LoBue C , Wilmoth K , Cullum CM , et al. Traumatic brain injury history is associated with earlier age of onset of frontotemporal dementia. J Neurol Neurosurg Psychiatry. 2016;87(8):817‐820. doi:10.1136/jnnp-2015-311438 26359171 PMC4835269

[11] Golimstok A , Cámpora N , Rojas JI , et al. Cardiovascular risk factors and frontotemporal dementia: a case‐control study. Transl Neurodegener. 2014;3:13. doi:10.1186/2047-9158-3-13 24995127 PMC4080770

[12] Katisko K , Solje E , Koivisto AM , et al. Prevalence of immunological diseases in a Finnish frontotemporal lobar degeneration cohort with the C9orf72 repeat expansion carriers and non‐carriers. J Neuroimmunol. 2018;321:29‐35. doi:10.1016/j.jneuroim.2018.05.011 29957385

[13] Borroni B , Alberici A , Agosti C , Premi E , Padovani A . Education plays a different role in frontotemporal dementia and Alzheimer's disease. Int J Geriatr Psychiatry. 2008;23(8):796‐800. doi:10.1002/gps.1974 18172915

[14] Rascovsky K , Hodges JR , Knopman D , et al. Sensitivity of revised diagnostic criteria for the behavioural variant of frontotemporal dementia. Brain. 2011;134(Pt 9):2456‐2477. doi:10.1093/brain/awr179 21810890 PMC3170532

[15] Gorno‐Tempini ML , Hillis AE , Weintraub S , et al. Classification of primary progressive aphasia and its variants. Neurology. 2011;76(11):1006‐1014. doi:10.1212/WNL.0b013e31821103e6 21325651 PMC3059138

[16] Litvan I , Agid Y , Calne D , et al. Clinical research criteria for the diagnosis of progressive supranuclear palsy (Steele‐Richardson‐Olszewski syndrome): report of the NINDS‐SPSP international workshop. Neurology. 1996;47(1):1‐9. doi:10.1212/wnl.47.1.1 8710059

[17] Armstrong MJ , Litvan I , Lang AE , et al. Criteria for the diagnosis of corticobasal degeneration. Neurology. 2013;80(5):496‐503. doi:10.1212/WNL.0b013e31827f0fd1 23359374 PMC3590050

[18] McKhann GM , Knopman DS , Chertkow H , et al. The diagnosis of dementia due to Alzheimer's disease: recommendations from the National Institute on Aging‐Alzheimer's Association workgroups on diagnostic guidelines for Alzheimer's disease. Alzheimers Dement. 2011;7(3):263‐269. doi:10.1016/j.jalz.2011.03.005 21514250 PMC3312024

[19] Kaivorinne AL , Krüger J , Udd B , Majamaa K , Remes AM . Mutations in CHMP2B are not a cause of frontotemporal lobar degeneration in Finnish patients. Eur J Neurol. 2010;17(11):1393‐1395. doi:10.1111/j.1468-1331.2010.03028.x 20412296

[20] Kaivorinne AL , Krüger J , Kuivaniemi K , et al. Role of MAPT mutations and haplotype in frontotemporal lobar degeneration in northern Finland. BMC Neurol. 2008;8:48. doi:10.1186/1471-2377-8-48 19091059 PMC2625345

[21] Krüger J , Kaivorinne AL , Udd B , Majamaa K , Remes AM . Low prevalence of progranulin mutations in Finnish patients with frontotemporal lobar degeneration. Eur J Neurol. 2009;16(1):27‐30. doi:10.1111/j.1468-1331.2008.02272.x 19049508

[22] Goldman JS , Farmer JM , Wood EM , et al. Comparison of family histories in FTLD subtypes and related tauopathies. Neurology. 2005;65(11):1817‐1819. doi:10.1212/01.wnl.0000187068.92184.63 16344531

[23] Gossink F , Dols A , Stek ML , et al. Early life involvement in C9orf72 repeat expansion carriers. J Neurol Neurosurg Psychiatry. 2022;93(1):93‐100. Published online April 27, 2021:jnnp‐2020‐325994. doi:10.1136/jnnp-2020-325994 33906932

[24] Bede P , Siah WF , McKenna MC , S LHS . Consideration of C9orf72‐associated ALS‐FTD as a neurodevel‐opmental disorder: insights from neuroimaging. J Neurol Neurosurg Psychiatry. 2020;91(11):1138. doi:10.1136/jnnp-2020-324416 32855286

[25] Livingston G , Huntley J , Sommerlad A , et al. Dementia prevention, intervention, and care: 2020 report of the lancet commission. Lancet. 2020;396(10248):413‐446. doi:10.1016/S0140-6736(20)30367-6 32738937 PMC7392084

[26] Atkins ER , Bulsara MK , Panegyres PK . Cerebrovascular risk factors in early‐onset dementia. J Neurol Neurosurg Psychiatry. 2012;83(6):666‐667. doi:10.1136/jnnp.2009.202846 20639379

[27] De Reuck JL , Deramecourt V , Cordonnier C , Leys D , Pasquier F , Maurage CA . Cerebrovascular lesions in patients with frontotemporal lobar degeneration: a neuropathological study. Neurodegener Dis. 2012;9(4):170‐175. doi:10.1159/000335447 22377662

[28] Miller ZA , Rankin KP , Graff‐Radford NR , et al. TDP‐43 frontotemporal lobar degeneration and autoimmune disease. J Neurol Neurosurg Psychiatry. 2013;84(9):956‐962. doi:10.1136/jnnp-2012-304644 23543794 PMC3840954

[29] Torralva T , Sposato LA , Riccio PM , et al. Role of brain infarcts in behavioral variant frontotemporal dementia: clinicopathological characterization in the National Alzheimer's coordinating center database. Neurobiol Aging. 2015;36(10):2861‐2868. doi:10.1016/j.neurobiolaging.2015.06.026 26220367 PMC4562890

[30] Katisko K , Haapasalo A , Koivisto A , et al. Low prevalence of cancer in patients with frontotemporal lobar degeneration. J Alzheimers Dis. 2018;62(2):789‐794. doi:10.3233/JAD-170854 29480183

[31] Westeneng HJ , van Veenhuijzen K , van der Spek RA , et al. Associations between lifestyle and amyotrophic lateral sclerosis stratified by C9orf72 genotype: a longitudinal, population‐based, case‐control study. Lancet Neurol. 2021;20(5):373‐384. doi:10.1016/S1474-4422(21)00042-9 33894192

